# Identification of plasticity and interactions of a highly conserved motif within a picornavirus capsid precursor required for virus infectivity

**DOI:** 10.1038/s41598-019-48170-9

**Published:** 2019-08-13

**Authors:** Thea Kristensen, Graham J. Belsham

**Affiliations:** 1 DTU National Veterinary Institute, Lindholm, Kalvehave, 4771 Denmark; 2 Present Address: Department of Veterinary and Animal Sciences, University of Copenhagen, Grønnegårdsvej 15, 1870 Frederiksberg C, Denmark

**Keywords:** Microbiology, Molecular biology, Virus structures, Chaperones, Microbiology

## Abstract

The picornavirus family includes poliovirus (PV) (genus: *enterovirus*), human rhinoviruses (*enterovirus*) and foot-and-mouth disease virus (FMDV) (*aphthovirus*). These are responsible for important human and animal health concerns worldwide including poliomyelitis, the common cold and foot-and-mouth disease (FMD) respectively. In picornavirus particles, the positive-sense RNA genome (ca. 7–9 kb) is packaged within a protein shell (capsid) usually consisting of three surface exposed proteins, VP1, VP2 and VP3 plus the internal VP4, which are generated following cleavage of the capsid precursor by a virus-encoded protease. We have previously identified a motif near the C-terminus of FMDV VP1 that is required for capsid precursor processing. This motif is highly conserved among other picornaviruses, and is also likely to be important for their capsid precursor processing. We have now determined the plasticity of residues within this motif for virus infectivity and found an important interaction between FMDV residue VP1 R188 within this conserved motif and residue W129 in VP2 that is adjacent in the virus capsid. The FMDV (VP1 R188A) mutant virus has only been rescued with the secondary substitution VP2 W129R. This additional change compensates for the defect resulting from the VP1 R188A substitution and restored both capsid precursor processing and virus viability.

## Introduction

**Figure 1:**
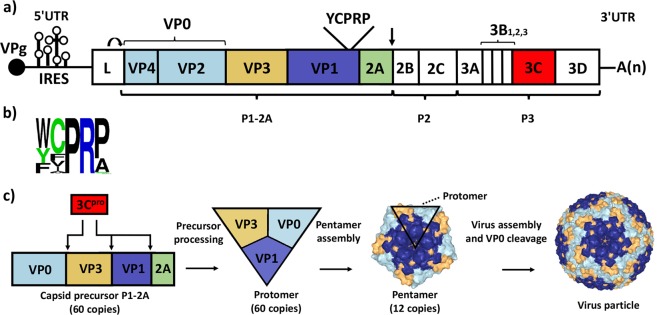
Picornavirus capsid assembly. (**a**) The genome organization of FMDV is shown. The viral RNA encodes a large polyprotein that is processed to yield precursors and finally mature products. The FMDV capsid protein precursor (P1-2A) includes the conserved YCPRP motif near the C-terminus of VP1. The Leader protease (L^pro^) cleaves itself from the P1-2A precursor. Ribosomal skipping at the C-terminus of 2A is marked with an arrow. The 3C^pro^ (red) is responsible for cleavage of all other junctions. (**b**) A logoplot showing variation in this conserved motif amongst a collection of picornavirus sequences (from http://www.virology.wisc.edu/acp/Aligns/aligns/picorna.p1) was generated using https://weblogo.berkeley.edu. (**c**) The pathway of capsid assembly is shown from the processing of the capsid precursor P1-2A by the 3C^pro^, to the formation of protomers, the assembly of pentamers and finally the generation of complete capsids. The cleavage of VP0 to VP4 and VP2 occurs during the formation of virus particles. The structures of the pentamer and virus particle are from PDB 4GH4^21^.

Picornavirus RNA genomes include a single, large, open reading frame, ca. 6000–7000 nt long, encoding a polyprotein that is co- and post-translationally processed to multiple precursors and then mature virus proteins^1^. Three or four primary products are made, namely the Leader protein (in many picornaviruses), the capsid precursor (P1 or P1-2A) and precursors of the non-structural proteins P2 and P3, (Fig. 1a). The structural and non-structural protein precursors rapidly separate. In *cardio*- and *aphthoviruses*, breakage of the polypeptide chain at the 2A/2B junction occurs during translation^2,3^. The 2A remains part of the capsid precursor as P1-2A until removed by the 3C protease (3C^pro^), (Fig. 1a,c). In *enteroviruses*, the 2A protease cleaves the VP1/2A junction to release P1^4,5^.

The junction between P2 and P3 plus their internal protein junctions are cleaved by 3C^pro^, to produce the various non-structural proteins. In *aphtho-* and *cardioviruses*, the 3C^pro^ also processes the P1-2A precursor into VP0, VP3 and VP1 plus 2A^6,7^. However, the enterovirus P1 precursor is cleaved by 3CD protease (3CD^pro^)^8,9^. Following cleavage of the capsid precursor, VP0, VP3 and VP1 remain together as a protomer, (Fig. 1c). Five protomers combine to form a pentamer and 12 pentamers assemble to make virus particles, (Fig. 1c). In most picornaviruses, VP0 is cleaved to VP2 and VP4 during particle assembly.

Protein functionality is highly dependent on correct folding. Evidence suggests that cellular chaperones, including different heat shock proteins (e.g. Hsp90), are required for assembly of various picornaviruses^10,11^. Hsp90 protects the PV capsid precursor from degradation by proteasomes, which remove misfolded proteins^10^.

We showed previously that inhibiting the cleavage of individual junctions within the FMDV P1-2A capsid precursor, did not affect cleavage of other junctions within the precursor^12^. However, we have also identified a short motif within the C-terminus of FMDV VP1 which is critical for processing of the entire capsid precursor^13^. This motif, YCPRP (residues 185–189), is highly conserved amongst FMDVs of all seven serotypes, even though VP1 is the most variable FMDV protein with only 26% of its residues being invariant^14–16^. The motif is also highly conserved in other picornaviruses (FCPRP in cardioviruses and WCPRP in enteroviruses), (Fig. 1a,b). In cells expressing the FMDV P1-2A precursor with 3C^pro^, we showed that substituting single residues within this motif severely inhibited cleavage at the VP0/VP3 and the VP3/VP1 junctions. These junctions are far away from the modified motif in the linear sequence suggesting these changes affect the overall structure of the capsid precursor^13^. Earlier studies had shown that removal of the VP1 C-terminus from the capsid precursor of both PV and FMDV, which deleted this conserved motif, resulted in truncated precursors that could not be processed *in vitro* whilst retaining the unmodified VP0/VP3 and VP3/VP1 junctions^17,18^. Thus, it seems that the conserved motif is required for capsid precursor processing in multiple members of the picornavirus family.

We have now defined the plasticity of this motif within FMDV and identified adaptation, during mutant virus rescue, which revealed interaction between this motif and the adjacent VP2 molecule.

## Results

### Production of FMDVs with modifications in the highly conserved YCPRP motif

We have now investigated the effect of modifying the conserved YCPRP motif within the context of infectious FMDVs. Modifications were introduced into a full-length infectious cDNA based on the O1 Kaufbeuren FMDV but with the coding region for VP2-VP3-VP1-2A derived from the A22 Iraq virus (as used previously^12,13,19,20^). The plasmids were named according to their modifications: FMDV (VP1 ∆185–190), FMDV (VP1 Y185A), FMDV (VP1 C186A), FMDV (VP1 P187A), FMDV (VP1 R188A) and FMDV (VP1 P189A). In order to rescue the viruses, full-length RNA transcripts were produced *in vitro*, using T7 RNA polymerase, and introduced into BHK cells by electroporation. Samples from the harvested cells were added to fresh cells (passage 1). Already at this first passage, the FMDV (wt) and the mutants FMDV (VP1 C186A) and FMDV (VP1 189A) produced CPE in the cells (Table 1, as described previously^13^). At the second passage, the mutant FMDV (VP1 R188A) also produced some CPE and at the third passage, this mutant produced full CPE. None of the other mutant transcripts (FMDV (VP1 ∆185–190), FMDV (VP1 Y185A) or FMDV (VP1 P187A)) produced CPE even after four passages (Table 1).

**Table 1:** The indicated RNA transcripts were introduced into BHK cells using electroporation. After 24 hr, the cells and medium were frozen and an aliquot of 800 µl was added to fresh cells (passage 1). After CPE developed, or 48 hr (whichever was sooner), the procedure was repeated for passages 2, 3 and 4, however in cases of full CPE the virus aliquot for the next passage was reduced to 200 µl. Full CPE is indicated as +++, some CPE is indicated as+, while the absence of CPE is indicated as -, n.d. means not determined and Psg. indicates the passage number. “Repetition” indicates that a new synthesis and introduction of the RNA into cells was performed.

RNA transcript	CPE at Psg. 1	CPE at Psg. 2	CPE at Psg. 3	CPE at Psg. 4	Changes in rescued virus	Comment
FMDV (VP1 ∆185–189)	−	−	−	−		
FMDV (VP1 Y185A)	−	−	−	−		
FMDV (VP1 C186A)	+++	+++	+++	n.d.	−	
FMDV (VP1 P187A)	−	−	−	−		
FMDV (VP1 R188A)	−	+	+++	+++	VP2 W129R + VP3 E70G	FMDV (VP1 R188A)*
FMDV (VP1 P189A)	+++	+++	+++	n.d.	−	
FMDV (VP2 W129R)	+++	+++	+++	n.d.	−	
FMDV (VP3 E70G)	+++	+++	+++	n.d.	−	
FMDV (VP1 Y185A) repetition	−	−	−	−		
FMDV (VP1 Y185A + VP2 W129R)	−	−	−	−		
FMDV (VP1 Y185A + VP3 E70G)	−	−	−	−		
FMDV (VP1 R188A) repetition	−	+	+++	+++	VP2 W129R + VP2 A74G^‡^	FMDV (VP1 R188A)**
FMDV (VP1 R188A + VP2 W129R)	+++	+++	+++	n.d.	−	
FMDV (VP1 R188A + VP3 E70G)	−	−	−	−		
FMDV (wt)	+++	+++	+++	n.d.	−	
None	−	−	−	−		

^‡^: In 12/15 clones sequenced the VP2 A74G change was present, while in 1/15 clones the substitution F75L was also present, the VP2 W129R change alone was present in 3/15 clones.

RNA was extracted from virus harvests when CPE had been observed and RT-PCR was performed to amplify the P1-2A coding region within multiple, overlapping, amplicons that were sequenced. The wt virus sequence was unchanged and the rescued FMDV (VP1 C186A) and FMDV (VP1 P189A) had retained their encoded amino acid substitutions. For both of these mutants, no secondary mutations were observed within the P1-2A coding sequence. However, the rescued FMDV (VP1 R188A) mutant had acquired two secondary mutations. These encoded the substitutions VP2 W129R and VP3 E70G (Table 1) that are located far away (495 or 336 residues, respectively) from the conserved YCPRP motif in the linear amino acid sequence. This mutant virus, with the additional substitutions, is termed FMDV (VP1 R188A*) to indicate that it has additional changes. The delayed appearance of CPE during the rescue of this mutant is consistent with the apparent need for virus adaptation, with the acquisition of additional compensatory substitutions, to allow efficient virus growth.

### Identification of plasticity within the YCPRP motif

**Figure 2:**
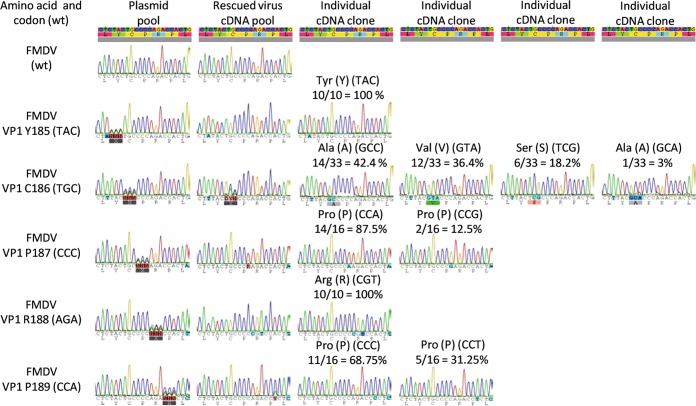
The plasticity of the YCPRP motif within infectious FMDV. Mutagenesis was performed on defective mutant full-length FMDV cDNA templates (see text) to modify the codon for each residue within the YCPRP individually to all possible codons (NNN). The pools of plasmids obtained were sequenced and the heterogeneity in each pool is shown. RNA transcripts were prepared from each mutant pool separately and introduced into cells to recover infectious virus. CPE was observed at passage 1 (c.f. the mutant template) in each case and then amplified through 2 passages in total. RNA was extracted from these rescued viruses, converted to cDNA and sequenced throughout the P1-2A coding region. No secondary substitutions were observed and heterogeneity was only observed within the sequence encoding the conserved motif in VP1. From each cDNA pool, a cDNA fragment, encoding VP1-2A, was amplified and inserted into a TOPO vector and individual clones (10–33) were sequenced. Representatives of the distinct individual sequences are shown together with the encoded amino acid sequence. The number and frequency (%) of each codon within the individual clones analyzed is shown. Note, within the rescued viruses, the presence of 4 distinct codons (encoding 3 different amino acids) at the position of C186, whereas only the wt TAC sequence (encoding Y) was present at the codon for residue 185. At the codons for residues P187, R188 and P189, only viruses with the wt amino acid were rescued but alternate (non-wt) codons were present.

To investigate which amino acid changes can be tolerated within the conserved YCPRP motif of the virus, the codons for each of its 5 amino acids were individually changed to the sequence NNN (corresponding to all four nucleotides at each position), to produce every possible codon. It should be noted that the template used for mutagenesis, in each case, was a mutant that failed to yield infectious virus at first passage. Plasmid pools, containing the degenerate codons encoding each residue, were produced and sequenced (as a pool) to demonstrate the heterogeneity at the modified codons (Fig. 2). RNA transcripts were then produced and introduced into BHK cells, as above. All of the mutant transcripts produced some CPE at the first passage and full CPE at the second passage, thus the mutagenesis had enhanced infectivity.

No secondary mutations were observed in the P1-2A coding sequence of any of the rescued NNN- mutant viruses outside of the targeted codon. For FMDV (VP1 Y185NNN) and FMDV (VP1 R188NNN) unique sequence was present in the rescued virus pool at the modified codon (Fig. 2). However, sequence heterogeneity was observed at the other modified codons, i.e. FMDV (VP1 C186NNN), FMDV (VP1 P187NNN) and FMDV (VP1 P189NNN) (Fig. 2). To identify these codons individually, amplicons containing the coding region for VP1-2A were cloned and plasmids from >10 individual colonies were sequenced. Each colony contains the sequence corresponding to the VP1-2A coding region from a single FMDV RNA genome. Sequences from the plasmid pool and each of the various clones from the FMDV cDNA were determined (Fig. 2) thus, this analysis provided an overview of the heterogeneity of the virus population and indicated which amino acids could be tolerated, within infectious virus, at each position (Fig. 2). Strikingly, there was a very strong selection for the tyrosine (Y) residue at position 185 with only the codon TAC, corresponding to the wt sequence, being observed at this site. In contrast, at residue 186, which in the wt sequence is a cysteine (C), a marked diversity of amino acid substitutions was apparent. Alanine (A), encoded by two different codons, valine (V) and serine (S) were observed at this site (Fig. 2). None of the rescued FMDV genomes derived from the FMDV (VP1 C186NNN) mutant encoded the wt C at this position. The identity of this residue appears to be relatively unconstrained, indeed, it was previously shown to be of little importance for the processing of the capsid precursor^13^. Furthermore, this residue shows the greatest diversity among the different picornaviruses (Fig. 1b). However, the observed residues encoded by the rescued FMDV (VP1 C186NNN) mutants all have a neutral charge and are relatively small. At residue 187, the rescued viruses only encoded a proline (P), as in the wt sequence, however, two synonymous codons (CCA and CCG) were observed, in contrast to the wt codon, (CCC). From the FMDV (VP1 R188NNN) mutant, only an arginine (R) residue, as present in the wt virus, was found. However, the codon (CGT) was different from the wt sequence (AGA) (Fig. 2). There was also strong selection for P at residue 189. Two synonymous codons (CCC and CCT) were observed, in contrast to the wt codon (CCA) (Fig. 2).

The strict requirement for the FMDV (VP1 Y185) and FMDV (VP1 R188) were fully consistent with our earlier studies where we showed that neither the P1-2A (VP1 Y185A) nor the P1-2A (VP1 R188A) capsid precursors could be processed^13^ and with the failure to rescue FMDV (VP1 Y185A) and FMDV (VP1 R188A) (without adaptations), (Table 1). Thus, residues VP1 Y185 and R188 are of high importance both for the processing of the FMDV capsid precursor and for virus viability. There was also a strong selection for both the wt VP1 P187 and VP1 P189 residues. These residues are not critical for the processing of the capsid precursor in the transient expression assay^13^. However, the FMDV (VP1 P187A) mutant could not be rescued (Table 1). In contrast, the FMDV (VP1 P189A) mutant was viable, indicating that alternative residues can be tolerated (see^16^). Thus, from all the possible versions of the motif (when changing one residue at a time), all the rescued FMDVs encoded the sequence YxPRP (where x can be at least C, A, V and S) consistent with the high degree of conservation of this motif among FMDVs. The YxPRP residues are very conserved among other picornaviruses (Fig. 1b) and thus it seems very likely that this motif plays a similar, important role in these other viruses.

### Rescue of mutant FMDVs containing secondary substitutions

Two secondary mutations were observed in the rescued FMDV (VP1 R188A*) mutant, namely VP2 W129R and VP3 E70G. Our earlier studies^13^ had shown that the VP1 R188A substitution in the FMDV capsid precursor P1-2A, when expressed in a transient expression assay, severely inhibited processing of the junctions between the structural proteins. Thus, since the FMDV (VP1 R188A*) mutant was clearly viable, we expected that one or both of the observed secondary mutations would have a positive effect on the cleavage of the junctions within the capsid precursor. To investigate the effect of these secondary mutations, each was introduced individually into the full-length FMDV (wt) cDNA and called FMDV (VP2 W129R) and FMDV (VP3 E70G) respectively. Furthermore, to determine how these two substitutions affected the properties of the non-processable mutant with the VP1 R188A substitution, the changes were also introduced into the quasi-infectious FMDV (VP1 R188A) cDNA clone. These plasmids were called FMDV (VP1 R188A + VP2 W129R) and FMDV (VP1 R188A + VP3 E70G). To analyze whether one of these secondary mutations also affected the viability of the FMDV (VP1 Y185A) mutant, each of the changes was also introduced into the non-infectious FMDV (VP1 Y185A) cDNA clone. These plasmids are referred to as FMDV (VP1 Y185A + VP2 W129R) and FMDV (VP1 Y185A + VP3 E70G). The rescue of viruses from FMDV (VP1 Y185A) and the FMDV (VP1 R188A) plasmids was repeated, in parallel, to confirm that no virus could be rescued from the FMDV (VP1 Y185A) mutant and to determine whether we could again rescue mutant virus from the quasi-infectious FMDV (VP1 R188A) mutant.

Following introduction of RNA transcripts into cells, the FMDV (VP2 W129R), FMDV (VP3 E70G) and the FMDV (VP1 R188A + VP2 W129R) mutants, all produced full CPE at the first passage (Table 1). As observed initially, the FMDV (VP1 R188A) only generated some signs of CPE at the second passage and full CPE was apparent at the third passage. This independently rescued virus is referred to as FMDV (VP1 R188A**). In contrast, no CPE was observed for the FMDV (VP1 R188A + VP3 E70G) mutant after four passages, (Table 1). Similarly, the attempts to rescue virus from the FMDV (VP1 Y185A), the FMDV (VP1 Y185A + VP2 W129R) and the FMDV (VP1 Y185A + VP3 E70G) transcripts were each unsuccessful since no CPE was seen in any of the four passages, (Table 1). Thus, the VP2 W129R substitution was able to compensate for the deleterious effect of the VP1 R188A modification on virus infectivity but could not compensate for the effect of the VP1 Y185A change.

To characterize the rescued viruses, RNA was isolated from cells displaying CPE and RT-PCR was performed to amplify the P1-2A coding region in multiple, overlapping amplicons, as above, and the sequence corresponding to the complete P1-2A precursor was determined. The rescued FMDV (VP2 W129R) and FMDV (VP3 E70G) had each retained their encoded amino acid substitutions and no secondary mutations were present within the P1-2A coding sequence, thus these substitutions did not have any significant deleterious effect on the wt virus. The FMDV (VP1 R188A + VP2 W129R) had also retained both substitutions and no additional sequence changes were observed within the P1-2A coding region. Sequencing of the independently rescued FMDV (VP1 R188A**) mutant, revealed that this virus had exactly the same secondary substitution (VP2 W129R) as observed in the FMDV (VP1 R188A*) mutant (Table 1). However, the VP3 E70G mutation, which was observed in the FMDV (VP1 R188A*) mutant, was not present in the FMDV (VP1 R188A**). Instead, another substitution was observed in VP2, namely A74G. This substitution was present in the majority of the virus (12/15 clones analyzed) whereas the rest of the viruses still had the wt sequence at this specific site (3/15). Furthermore, an additional substitution, VP2 F75L, was observed in just one of the clones obtained from the viral cDNA that also encoded the VP2 A74G substitution.

To investigate whether the secondary mutations VP2 W129R and VP3 E70G had any effect on virus growth, the virus yield from BHK cells infected with FMDV (VP2 W129R), FMDV (VP3 E70G) and the FMDV (VP1 R188A + VP2 W129R) mutants were determined in parallel with FMDV (wt). No major differences in their growth kinetics were observed (Supplementary Fig. S1).

### The effect of the secondary substitutions on P1-2A processing by 3C^pro^

**Figure 3:**
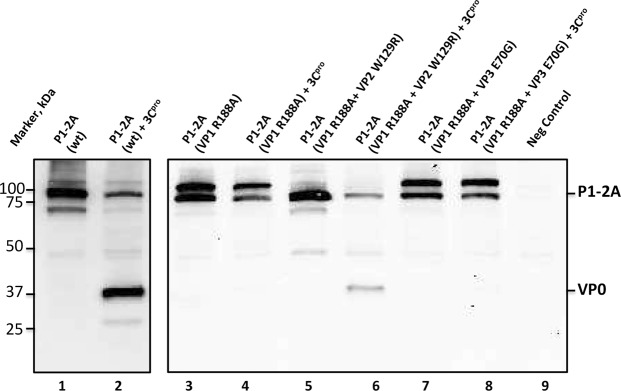
A second site substitution within VP2 compensates for the deleterious effect of the VP1 R188A modification in the FMDV P1-2A capsid precursor. Plasmids encoding wt or mutant forms of the FMDV P1-2A (as indicated) were expressed alone (odd numbered lanes) or with the FMDV 3C^pro^ (even numbered lanes) within BHK cells, as described in Methods section. Cell lysates were analyzed by SDS-PAGE and immunoblotting using guinea pig anti-FMDV O-Man antisera. Bound antibodies were visualized using the anti-guinea pig HRP-conjugated secondary antibodies and a chemiluminescence detection kit. The unprocessed P1-2A precursor and the VP0 processing product are indicated. Note the enhanced processing of the P1-2A (VP1 R188A) mutant in the presence of the substitution VP2 (W129R).

We also analyzed the effect of the two secondary substitutions, VP2 W129R and VP3 E70G, on the processing of the capsid precursor by 3C^pro^, both of these changes were introduced individually into the P1-2A (VP1 R188A) plasmids. These are referred to as P1-2A (VP1 R188A + VP2 W129R) and P1-2A (VP1 R188A + VP3 E70G). These plasmids together with the P1-2A (wt) and the P1-2A (VP1 R188A) were expressed in a transient expression assay either in the absence or presence of 3C^pro^, as described earlier^13^. As expected, when the wt plasmid was assayed alone it generated a product (P1-2A) of approximately 85 kDa (Fig. 3, lane 1). However, when co-expressed with 3C^pro^, the P1-2A (wt) product was processed and yielded a product of approximately 37 kDa (corresponding to VP0) (Fig. 3, lane 2). The P1-2A (VP1 R188A) and the P1-2A (VP1 R188A + VP3 E70G) both generated products of approximately 85 kDa corresponding to the capsid precursor in the absence of 3C^pro^ (Fig. 3, lanes 3 and 7). However, these mutants were highly resistant to cleavage in the presence of 3C^pro^ (Fig. 3, lanes 4 and 8). The P1-2A (VP1 R188A + VP2 W129R) mutant also made the capsid precursor when expressed alone (Fig. 3, lane 5) but, in contrast to these other mutants, in the presence of the 3C^pro^, a product corresponding to VP0 was also detected indicating that processing of the capsid precursor had occurred (Fig. 3, lane 6). It should be noted, however, that the production of VP0 from this mutant P1-2A appeared less efficient than from the processing of the wt capsid precursor (Fig. 3, lanes 2 and 6).

## Discussion

**Figure 4:**
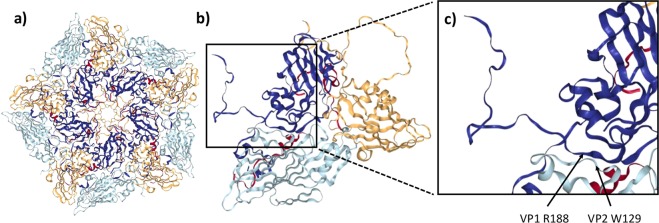
Location of residues VP1 R188 (within the conserved motif) and VP2 W129 in the virus structure. (**a**) The structure of a pentamer from within the FMDV A22 Iraq capsid is shown (from PDB 4GH4^21^). (**b**) An expanded view of a single protomer viewed (from a different angle) is shown and the box marks a further expanded view, as shown in (**c**) with the residues VP1 R188 and VP2 W129 at the interface between VP1 and VP2 indicated. VP1 is shown in dark blue and VP2 is in light blue.

**Figure 5:**
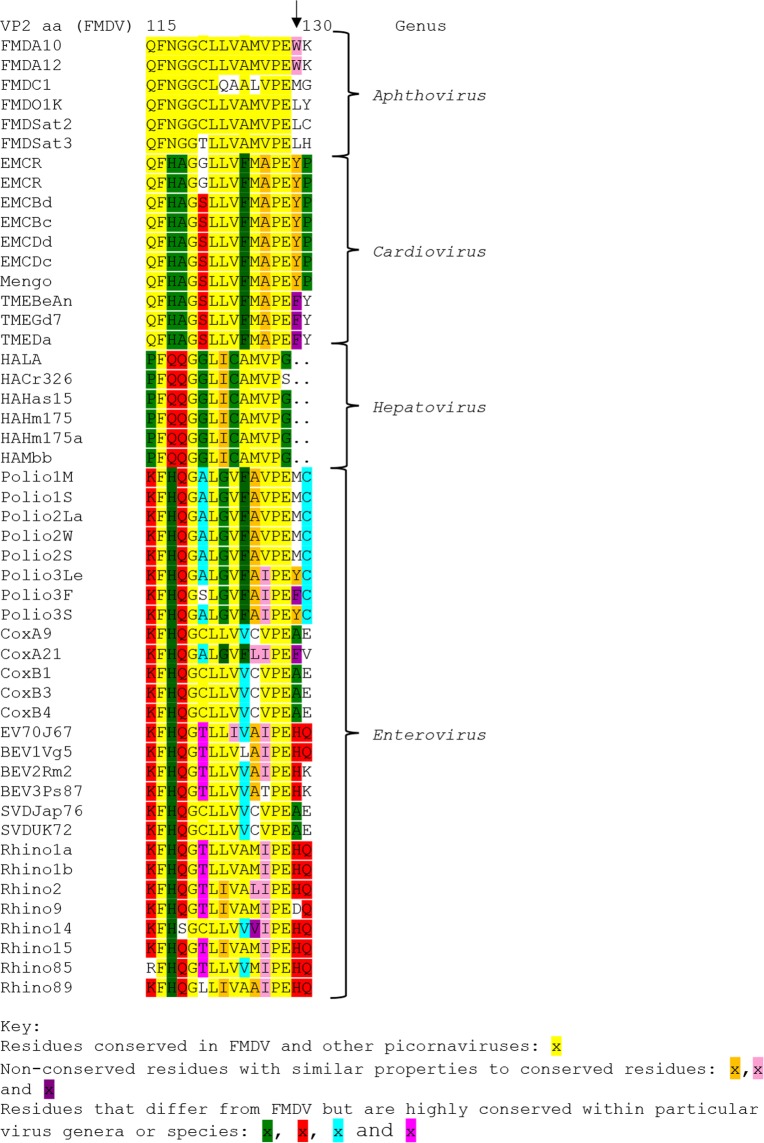
Conservation of VP2 sequences among picornaviruses. The amino acid sequence upstream of residue W129 within VP2 of FMDV (marked with an ↓) is highly conserved among other FMDVs and also among other picornaviruses. Amino acids that are identical among most FMDVs and are also conserved in other virus genera are highlighted in yellow. Non-conserved residues that have similar properties to the conserved residues are highlighted in other colours (see key). Residues that differ from FMDV but are highly conserved within particular virus genera or species are also marked. The selected viruses were derived from alignments available at (http://www.virology.wisc.edu/acp/Aligns/aligns/picorna.p1).

Recently, we showed that modifications of a short conserved motif (YCPRP) near the C-terminus of FMDV VP1, can prevent processing of the capsid precursor P1-2A at distal sites^13^. The VP0/VP3 and the VP3/VP1 cleavage sites are located far away (ca. 400 and almost 200 residues, respectively) from the modified motif in the linear sequence. Thus, it seems likely that the overall protein conformation has changed following modification of this motif consistent with the need for chaperones for capsid assembly^10,11^. The motif is very highly conserved among FMDV strains^13,16^ and is also well conserved among other members of the picornavirus family^13^. We have now explored, in detail, the impact of modifications within the conserved motif on virus infectivity. Deletion of the whole YCPRP motif or just the VP1 Y185A substitution blocked the production of viable virus. This was consistent with our previous study^13^, where we showed that the P1-2A precursors with these modifications, or with the VP1 R188A substitution, could not be processed by 3C^pro^. However, interestingly, the FMDV (VP1 R188A) mutant produced CPE during virus rescue that was delayed compared to the wt. When the rescued virus, termed FMDV (VP1 R188A*), was sequenced it was found that it had adapted and acquired two secondary substitutions, namely VP2 W129R and VP3 E70G. Repeating the electroporation and passage of the FMDV (VP1 R188A) again resulted in delayed CPE and sequencing of this rescued FMDV (VP1 R188A**) revealed that it had also acquired the substitution VP2 W129R. Furthermore, a major proportion (12/15 genomes analyzed) of this rescued FMDV (VP1 R188A**) had also acquired the secondary substitution VP2 A74G, and one of these also had the VP2 F75L substitution. These secondary mutations are located far away from the modified motif in the linear sequence. However, examination of the three-dimensional structure of FMDV A22^21^ revealed that VP1 R188 (within the conserved YCPRP motif) and VP2 W129 are located adjacent to each other at the interface between VP1 and VP2, (Fig. 4). The residues VP2 A74, VP2 F75L and VP3 E70 are located further away from the conserved motif in the virus structure, (Supplementary Fig. S2). Thus, it seems that there is an important interaction between residues VP1 R188 and VP2 W129. This interaction, and maybe the other substituted residues, may also be important for the folding of the capsid precursor (P1-2A). Surprisingly, the FMDV (VP1 R188A + VP3 E70G) did not yield any viable virus; it seems the VP2 W129R adaptation did not occur. The VP2 W129 residue is not conserved in other picornaviruses or even among other FMDV serotypes. However, immediately upstream of the VP2 W129 residue, there is a sequence QFNGGCLLVAMVPE (residues 115–128), which is very conserved among FMDVs (Fig. 5). Furthermore, this sequence is also highly conserved among other picornaviruses (Fig. 5). In the mature virus capsid, this sequence forms a beta strand that lies adjacent to the VP1 beta strand that ends in the conserved YCPRP motif. Substituting the R in the YCPRP motif, resulted in selection of mutant viruses with a secondary change VP2 W129R in two independently rescued viruses. Thus, it seems that there is a need for at least one positively charged amino acid at one of these locations. Among different FMDVs, the residue VP2 129 can be W, L or M, whereas in cardioviruses and polioviruses the corresponding residue is Y, F or M (Fig. 5). Thus, it seems that aromatic/hydrophobic residues are strongly selected at this site. The FMDV VP2 W129R substitution observed in the rescued FMDV (VP1 R188A*) and FMDV (VP1 R188A**) mutants is therefore unusual, in that the positively charged R residue is not normally observed at this position. This substitution is, most likely, selected due to the loss of the positive charge in the VP1 R188A mutant. It is, however, noteworthy that the VP2 W129R substitution alone was tolerated in infectious FMDV without any additional substitutions.

The FMDV (VP1 R188A + VP2 W129R) mutant generated CPE at the first passage and did not display a major defect in growth rate compared to the FMDV (wt) (Supplementary Fig. S1). This indicated that the VP2 W129R substitution is able to compensate efficiently for the deleterious effect of the VP1 R188A substitution in relation to virus viability. However, from the analysis of P1-2A processing (Fig. 3), it appears that the VP2 W129R substitution does not fully restore the processing efficiency of the mutant capsid precursor. In the presence of low levels of 3C^pro^, VP0 was generated less efficiently from the P1-2A (VP1 R188A + VP2 W129R) mutant than from the P1-2A (wt), (Fig. 3, lanes 2 and 6). However, it should be noted that the FMDV A22 Iraq strain, as used in this study, generates a lot of empty capsids within infected cells^12,20,22^, thus it seems that the virus produces an excess of capsid proteins, which are not required for virus production. Furthermore, we have shown previously that slowing processing at the VP0/VP3 junction, did not affect the growth rate of a mutant FMDV A22 Iraq with this defect^12^. It is noteworthy that in the transient expression assay, there is a fixed ratio for the level of the P1-2A precursor and the 3C^pro^. In contrast, within cells infected with FMDV, there is, overtime, an increasing level of the 3C^pro^ compared to the P1-2A precursor substrate, as the precursor is processed without loss of 3C^pro^. This may allow efficient processing of a slightly sub-optimal substrate within infected cells.

Interestingly, the FMDV (VP1 Y185A + VP2 W129R), FMDV (Y185A + VP3 E70G) and the FMDV (VP1 R188A + VP3 E70G) mutants could not be rescued even after four passages (Table 1). This indicates that the VP2 W129R substitution was unable to compensate for the deleterious VP1 Y185A substitution. This likely reflects that there is no direct interaction between VP1 Y185 and VP2 W129, c.f. the interaction between the VP1 R188 and VP2 W129 residues. In conclusion, the analyses we have presented here and previously^13^, demonstrate the critical importance of the YCPRP motif near the C-terminus of VP1 in FMDV for P1-2A processing and virus viability. This is consistent with its high conservation among FMDVs and across other genera of the picornavirus family (Fig. 1b). The identification of a specific compensatory substitution (VP2 W129R) in the presence of the VP1 R188A substitution suggests an important interaction between these residues in the mature virus particle and also potentially during the process of capsid precursor assembly. As the motif is highly conserved among different picornaviruses^13,16^, the results obtained here with FMDV are very likely to be applicable to other members of this important virus family.

## Methods

### Plasmid constructions

Plasmid pO1K/A22 contains a full-length FMDV cDNA with the A22 Iraq capsid coding sequence within an FMDV O1K backbone^12,19,20^ and is referred to here as FMDV (wt). Seven individual modifications to the coding sequence for VP1 were made as described previously^13^ using this plasmid as template for the mutagenesis. The products were called: FMDV (VP1 Δ185-189), FMDV (VP1 Y185A), FMDV (VP1 C186A), FMDV (VP1 P187A), FMDV (VP1 188A) and FMDV (VP1 189A). Additional modifications were individually introduced into FMDV (wt), FMDV (VP1 R188A) and FMDV (VP1 Y185A) backgrounds, as required. These new constructs were named FMDV (VP2 W129R), FMDV (VP3 E70G), FMDV (VP1 R188A + VP2 W129R), FMDV (VP1 R188A + VP3 E70G), FMDV (VP1 Y185A + VP2 W129R) and FMDV (VP1 Y185A + VP3 E70G) according to the capsid protein and residues changed. To investigate the effect of each of the secondary mutation observed in the FMDV (VP1 R188A*) mutant on cleavage of the capsid precursor, these mutations were introduced into a plasmid only containing the coding sequence of the capsid precursor as described earlier^12,13^. The resultant plasmids were named according to their modifications, P1-2A (VP1 R188A), P1-2A (VP1 R188A + VP2 W129R) and P1-2A (VP1 R188A + VP3 E70G). All primers for generating the different constructs are listed in Supplementary Table S1.

### Identification of YCPRP motif plasticity within infectious FMDV

To investigate which amino acids can be tolerated in place of the VP1 residues Y185, C186, P187, R188 and P189, modifications using primers with all four nucleotides at each position of the codons, (i.e. NNN) were introduced. Mutant plasmids that did not readily generate infectious virus were used as the templates for the mutagenesis using primers listed in Supplementary Table S1, hence rescued viruses should predominantly be obtained from modified cDNAs. Plasmid FMDV (VP1 Y185A) was used to make the mutant pools FMDV (VP1 Y185NNN) and FMDV (VP1 C186NNN) while FMDV (VP1 P187A) was used for FMDV (VP1 P187NNN) and FMDV (VP1 R188A) was used for FMDV (VP1 R188NNN) plus FMDV (VP1 P189NNN). After transformation of *E. coli*, all colonies generated per mutant were pooled and amplified. The plasmid DNA pools (potentially containing all codons at each modified site) were purified and sequenced to confirm the heterogeneity at each modified codon, (Fig. 2).

### Rescue of modified FMDVs

Plasmids, containing the full-length (wt or mutant) FMDV cDNAs were linearized by digestion with *Hpa*I, purified and transcribed *in vitro* using the MEGAscript® T7 Transcription Kit (Thermo Fisher Scientific), as described by the manufacturer. An aliquot of each RNA was checked for integrity, DNase treated and introduced into BHK cells (in PBS) by electroporation^23^. The cells, were transferred to Eagle’s medium (8 ml) with 5% calf serum, and incubated with 5% CO_2_ at 37 °C. After 24 h, the cells and medium were harvested by freeze/thawing and an aliquot passaged onto fresh BHK cells. The procedure was repeated for subsequent passages. Up to four passages were performed for each RNA transcript, incubations were continued in each passage until complete CPE was observed or for 48 h at most. RNA was isolated from cells that displayed CPE (i.e. containing infectious virus) using the RNeasy Mini Kit (Qiagen). The RNA was reverse transcribed using random primers. For each reaction, a negative control, lacking the reverse transcriptase (-RT), was included to verify that the PCR products were produced from viral RNA and not from residual plasmid template.

### Sequencing of rescued FMDVs

The virus-derived cDNA was used to amplify four overlapping fragments (ca.1000 bp each), using primers listed in Supplementary Table S2, corresponding to the entire P1-2A coding region. The amplicons were purified and sequenced, as above, using primers listed in Supplementary Table S2. Heterogeneity was observed in the FMDV (VP1 R188A**) mutant and in most of the FMDV NNN mutants. For all of the FMDV NNN mutants, the VP1-2A coding region was amplified using primer 14TPN9_Fw and 14TPN14_Rev (Supplementary Table S2) while for the FMDV (VP1 R188A**) mutant, primers 14TPN5_Fw and 14TPN8_Rev were used, (Supplementary Table S2). The products were cloned using TOPO XL or XL2 (Thermo Fisher Scientific). From individual colonies (10 initially), plasmid DNA was purified and sequenced using the same two primers. Sequences were analyzed using Geneious 9.0.2 (Biomatters).

### Virus titrations and growth rate assays

Rescued viruses were titrated in BHK cells as described previously^12^. To determine virus growth rates, BHK cells were infected with the wt or selected mutant FMDVs using a MOI = 0.01, essentially as described previously^12^. The cells were incubated prior to harvesting at 0, 3, 6, 10 and 24 h p.i. and then frozen; virus harvests were titrated in fresh BHK cells and the virus yields (as TCID_50_/ml) were calculated.

### Transient expression assay and western blot

Transient expression assays and immunoblotting were performed as described previously^13^. For the blots, PBS containing 5% bovine serum albumin (BSA) and 0.1% Tween20 was used as blocking buffer and the guinea pig anti-FMDV O-Manisa antisera was used as primary antibody.

## Supplementary information

Supplementary information

## Data Availability

The authors confirm that the data supporting the findings of this study are available within the article and its supplementary materials.
