# Supplementary material for: Identification of plasticity and interactions of a highly conserved motif within a picornavirus capsid precursor required for virus infectivity
Source: Sci Rep. 2019 Aug 13;9:11747. doi: 10.1038/s41598-019-48170-9 (PMC6692319; doi:10.1038/s41598-019-48170-9)

Supplementary information for:

Identification of plasticity and interactions of a highly conserved motif within a picornavirus capsid precursor required for virus infectivity

Thea Kristensen[1] & Graham J. Belsham[1, 2]*

1: DTU National Veterinary Institute, Lindholm, Kalvehave 4771, Denmark

2: Current address, Department of Veterinary and Animal Sciences, University of Copenhagen, Grønnegårdsvej 15, 1870 Frederiksberg C, Denmark

Table S1: Primers used for site-directed mutagenesis

Primers for Megaprimers	Template	Change	Product Name
14TPN9_Fw: ATCCATGCTGAGTGGGACACAG<br>1PTK43_R: ACACCTCCACTGCCAACA**GG**AGTTCGGCACGCT*	FMDV (wt)	Deletion	FMDV (VP1 Δ185-189)
14TPN9_Fw: ATCCATGCTGAGTGGGACACAG<br>1PTK48_R: CAGTGGTCTGGGGCA**AGC**GAGTTCGGCA	FMDV (wt)	VP1 Y185A<br>(TAC-> AGC)	FMDV (VP1 Y185A)
14TPN9_Fw: ATCCATGCTGAGTGGGACACAG<br>1PTK49_R: CCAACAGTGGTCTGGG**AGC**GTAGAGTTC	FMDV (wt)	VP1 C186A<br>(TGC-> AGC)	FMDV (VP1 C186A)
14TPN9_Fw: ATCCATGCTGAGTGGGACACAG<br>1PTK50_R: ACTGCCAACAGTGGTCT**AGC**GCAGTAGA	FMDV (wt)	VP1 P187A<br>(CCC-> AGC)	FMDV (VP1 P187A)
14TPN9_Fw: ATCCATGCTGAGTGGGACACAG<br>1PTK51_R: CCACTGCCAACAGTGG**AGC**GGGGCAGTA	FMDV (wt)	VP1 R188A<br>(AGA-> AGC)	FMDV (VP1 R188A)
14TPN9_Fw: ATCCATGCTGAGTGGGACACAG<br>1PTK52_R: CCTCCACTGCCAACAG**AGC**TCTGGGGCA	FMDV (wt)	VP1 P189A<br>(CCA-> AGC)	FMDV (VP1 P189A)
14TPN9_Fw: ATCCATGCTGAGTGGGACACAG<br>1PTK56_R: CTTGAGACGACACCTC**AGC**TGCCAACAG	FMDV (wt)	VP1 V193A<br>(GTG-> AGC)	FMDV (VP1 V193A)
14TPN9_Fw: ATCCATGCTGAGTGGGACACAG<br>1PTK63_R: CAGTGGTCTGGGGCA**NNN**tAGTTCGGCA	FMDV (VP1 Y185A)	VP1 Y185<br>(TAC-> NNN)	FMDV (VP1 Y185NNN)
14TPN9_Fw: ATCCATGCTGAGTGGGACACAG<br>1PTK68_R: CCAACAGTGGTCTGGG**NNN**GTAaAGTTCGGCACGC	FMDV (VP1 Y185A)	VP1 C186<br>(TGC->NNN)	FMDV (VP1 C186NNN)
14TPN9_Fw: ATCCATGCTGAGTGGGACACAG<br>1PTK65_R: ACTGCCAAtAGTGGTCT**NNN**GCAGTAGA	FMDV (VP1 P187A)	VP1 P187<br>(CCC-> NNN)	FMDV (VP1 P187NNN)
14TPN9_Fw: ATCCATGCTGAGTGGGACACAG<br>1PTK66_R: CCACTGCCAAaAGTGG**NNN**GGGGCAGTA	FMDV (VP1 Y185A)	VP1 R188<br>(AGA-> NNN)	FMDV (VP1 R188NNN)
14TPN9_Fw: ATCCATGCTGAGTGGGACACAG<br>1PTK69_R: CCTCCACTGCCAAgAG**NNN**TCTGGGGCAGTAGA	FMDV (VP1 Y185A)	VP1 P189<br>(CCA-> NNN)	FMDV (VP1 P189NNN)
14TPN5_Fw: GAGTGTGGGAGTCACGTACG<br>1PTK73_R: GGTGAACTCTTT**CCG**TTCgGGGACCATGG	FMDV (wt),<br>(VP1 R188A)<br>(VP1 Y185A)<br>P1-2A (VP1 R188A)	VP2 W129R<br>(TGG->CGG)	FMDV (VP2 W129R)<br>FMDV (VP1 R188A + VP2 W129R)<br>FMDV (VP1 Y185A + VP2 W129R)<br>P1-2A (VP1 R188A + VP2 W129R)
14TPN7_Fw: GACAAATCAAGGTTTATGCCAACATTGC<br>1PTK75_R: GCCAGAAGACGCTG**CCC**GTCtGTTCTTGTC	FMDV (wt),<br>(VP1 R188A)<br>(VP1 Y185A)<br>P1-2A (VP1 R188A)	VP3 E70G<br>(GAG->GGG)	FMDV (VP3 E70G)<br>FMDV (VP1 R188A + VP3 E70G)<br>FMDV (VP1 Y185A + VP3 E70G)

Bold = codon change. *Deletion between the two bold nucleotides. Small letters = Synonymous mutation (tag).

Table S2: Primers for generating PCR products from cDNA and their sequencing

**Primers for PCR product and sequencing**
10PPN36_Fw: TGAATACAACTGACTGTTTTATCGC<br>14TPN6_Rev: CAAACAGGTGCTTCTTGAAAATCTTTC
14TPN5_Fw: GAGTGTGGGAGTCACGTACG<br>14TPN10_Rev: CTGCCACATCAGACGCAGTGT<br>**Additional primer for sequencing of this PCR product:**<br>14TPN8_Rev: CGTCCGAACAAGCGACCGGTA
14TPN7_Fw: GACAAATCAAGGTTTATGCCAACATTGC<br>14TPN12_Rev: GGAGCGCGAGCCTCGTAAAT
14TPN9_Fw: ATCCATGCTGAGTGGGACACAG<br>14TPN14_Rev: GTCGAGACCGGTTCTGATGGC

Figure S1. Growth rate of rescued wt and mutant viruses in BHK cells. The cells were infected using a MOI= 0.01 of each virus stock as indicated. After the indicated times, the viruses were harvested by freezing and the virus yield determined as $TCID_{50}$/ml in fresh BHK cells.

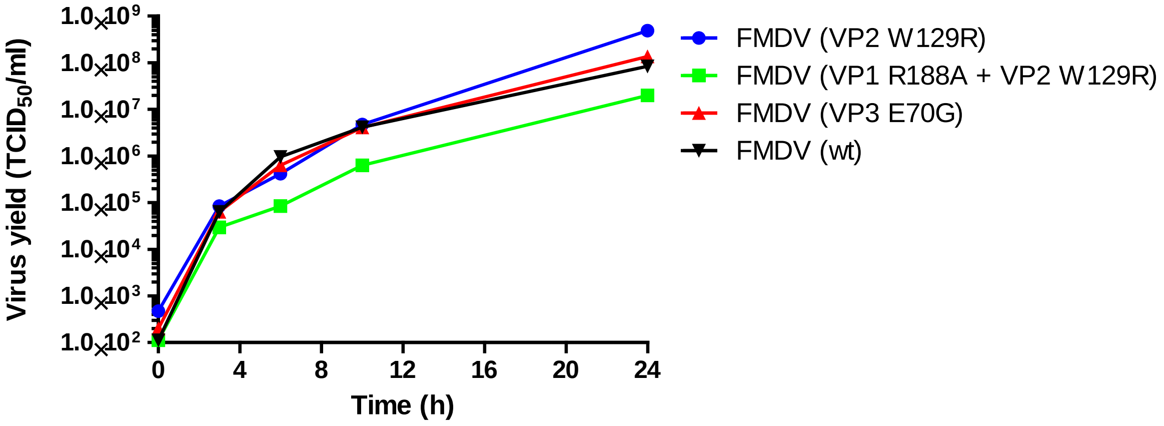

Figure S2. Location of second site changes detected in rescued viruses within the virus capsid. The locations of residues VP2 A74, VP2 F75, VP2 W129, VP3 E70 and VP1 R188 within the structure of the FMDV A22 Iraq pentamer (as described in PDB 4GH4[21]) are indicated.

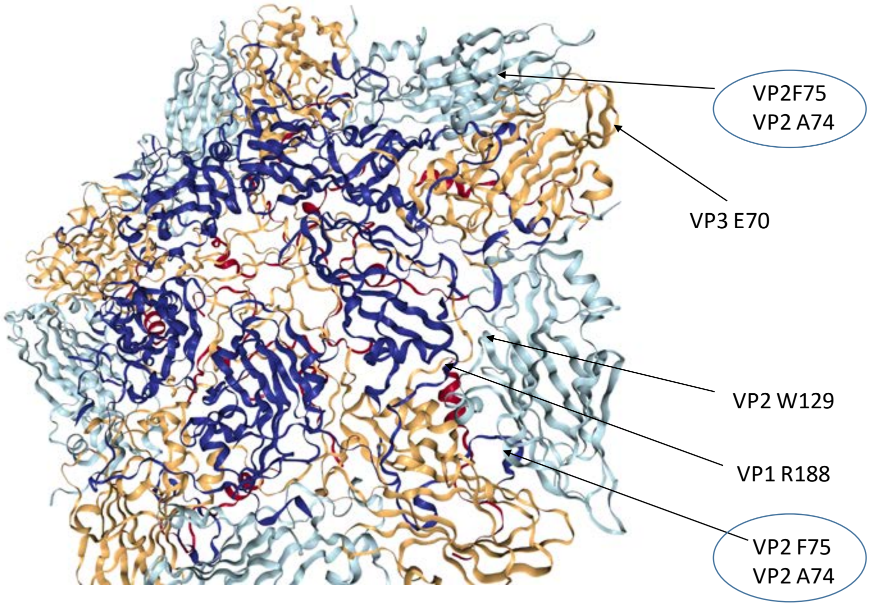

Supplement: Supplementary file 1 — Supplementary information [file 41598_2019_48170_MOESM1_ESM.pdf]
